# Broad targeting of angiogenesis for cancer prevention and therapy

**DOI:** 10.1016/j.semcancer.2015.01.001

**Published:** 2015-12

**Authors:** Zongwei Wang, Charlotta Dabrosin, Xin Yin, Mark M. Fuster, Alexandra Arreola, W. Kimryn Rathmell, Daniele Generali, Ganji P. Nagaraju, Bassel El-Rayes, Domenico Ribatti, Yi Charlie Chen, Kanya Honoki, Hiromasa Fujii, Alexandros G. Georgakilas, Somaira Nowsheen, Amedeo Amedei, Elena Niccolai, Amr Amin, S. Salman Ashraf, Bill Helferich, Xujuan Yang, Gunjan Guha, Dipita Bhakta, Maria Rosa Ciriolo, Katia Aquilano, Sophie Chen, Dorota Halicka, Sulma I. Mohammed, Asfar S. Azmi, Alan Bilsland, W. Nicol Keith, Lasse D. Jensen

**Affiliations:** a Department of Urology, Massachusetts General Hospital, Harvard Medical School, Boston, MA, USA; b Department of Oncology, Linköping University, Linköping, Sweden; c Department of Clinical and Experimental Medicine, Linköping University, Linköping, Sweden; d Medicine and Research Services, Veterans Affairs San Diego Healthcare System & University of California, San Diego, San Diego, CA, USA; e Lineberger Comprehensive Cancer Center, University of North Carolina, Chapel Hill, NC, USA; f Molecular Therapy and Pharmacogenomics Unit, AO Isituti Ospitalieri di Cremona, Cremona, Italy; g Department of Hematology and Medical Oncology, Emory University, Atlanta, GA, USA; h Department of Basic Medical Sciences, Neurosciences and Sensory Organs, University of Bari Medical School, Bari, Italy; i National Cancer Institute Giovanni Paolo II, Bari, Italy; j Department of Biology, Alderson Broaddus University, Philippi, WV, USA; k Department of Orthopedic Surgery, Arthroplasty and Regenerative Medicine, Nara Medical University, Nara, Japan; l Physics Department, School of Applied Mathematics and Physical Sciences, National Technical University of Athens, Athens, Greece; m Mayo Graduate School, Mayo Clinic College of Medicine, Rochester, MN, USA; n Department of Experimental and Clinical Medicine, University of Florence, Florence, Italy; o Department of Biology, College of Science, United Arab Emirate University, United Arab Emirates; p Faculty of Science, Cairo University, Cairo, Egypt; q Department of Chemistry, College of Science, United Arab Emirate University, United Arab Emirates; r University of Illinois at Urbana Champaign, Urbana, IL, USA; s School of Chemical and Bio Technology, SASTRA University, Thanjavur, India; t Department of Biology, University of Rome “Tor Vergata”, Rome, Italy; u Ovarian and Prostate Cancer Research Trust Laboratory, Guilford, Surrey, UK; v New York Medical College, New York City, NY, USA; w Department of Comparative Pathobiology, Purdue University Center for Cancer Research, West Lafayette, IN, USA; x School of Medicine, Wayne State University, Detroit, MI, USA; y Institute of Cancer Sciences, University of Glasgow, Glasgow, UK; z Department of Medical, and Health Sciences, Linköping University, Linköping, Sweden; A Department of Microbiology, Tumor and Cell Biology, Karolinska Institutet, Stockholm, Sweden

**Keywords:** Angiogenesis, Cancer, Phytochemicals, Treatment, Anti-angiogenic

## Abstract

Deregulation of angiogenesis – the growth of new blood vessels from an existing vasculature – is a main driving force in many severe human diseases including cancer. As such, tumor angiogenesis is important for delivering oxygen and nutrients to growing tumors, and therefore considered an essential pathologic feature of cancer, while also playing a key role in enabling other aspects of tumor pathology such as metabolic deregulation and tumor dissemination/metastasis. Recently, inhibition of tumor angiogenesis has become a clinical anti-cancer strategy in line with chemotherapy, radiotherapy and surgery, which underscore the critical importance of the angiogenic switch during early tumor development. Unfortunately the clinically approved anti-angiogenic drugs in use today are only effective in a subset of the patients, and many who initially respond develop resistance over time. Also, some of the anti-angiogenic drugs are toxic and it would be of great importance to identify alternative compounds, which could overcome these drawbacks and limitations of the currently available therapy. Finding “the most important target” may, however, prove a very challenging approach as the tumor environment is highly diverse, consisting of many different cell types, all of which may contribute to tumor angiogenesis. Furthermore, the tumor cells themselves are genetically unstable, leading to a progressive increase in the number of different angiogenic factors produced as the cancer progresses to advanced stages. As an alternative approach to targeted therapy, options to broadly interfere with angiogenic signals by a mixture of non-toxic natural compound with pleiotropic actions were viewed by this team as an opportunity to develop a complementary anti-angiogenesis treatment option. As a part of the “Halifax Project” within the “Getting to know cancer” framework, we have here, based on a thorough review of the literature, identified 10 important aspects of tumor angiogenesis and the pathological tumor vasculature which would be well suited as targets for anti-angiogenic therapy: (1) endothelial cell migration/tip cell formation, (2) structural abnormalities of tumor vessels, (3) hypoxia, (4) lymphangiogenesis, (5) elevated interstitial fluid pressure, (6) poor perfusion, (7) disrupted circadian rhythms, (8) tumor promoting inflammation, (9) tumor promoting fibroblasts and (10) tumor cell metabolism/acidosis. Following this analysis, we scrutinized the available literature on broadly acting anti-angiogenic natural products, with a focus on finding qualitative information on phytochemicals which could inhibit these targets and came up with 10 prototypical phytochemical compounds: (1) oleanolic acid, (2) tripterine, (3) silibinin, (4) curcumin, (5) epigallocatechin-gallate, (6) kaempferol, (7) melatonin, (8) enterolactone, (9) withaferin A and (10) resveratrol. We suggest that these plant-derived compounds could be combined to constitute a broader acting and more effective inhibitory cocktail at doses that would not be likely to cause excessive toxicity. All the targets and phytochemical approaches were further cross-validated against their effects on other essential tumorigenic pathways (based on the “hallmarks” of cancer) in order to discover possible synergies or potentially harmful interactions, and were found to generally also have positive involvement in/effects on these other aspects of tumor biology. The aim is that this discussion could lead to the selection of combinations of such anti-angiogenic compounds which could be used in potent anti-tumor cocktails, for enhanced therapeutic efficacy, reduced toxicity and circumvention of single-agent anti-angiogenic resistance, as well as for possible use in primary or secondary cancer prevention strategies.

## 1 Introduction to tumor angiogenesis

Vessel formation in both health and disease occur through either vasculogenesis – i.e. the recruitment of bone marrow-derived endothelial progenitor cells to form new vessels, angiogenesis – i.e. the sprouting and growth of new vessels from an existing vasculature or intussusception – i.e. the division or splitting of a blood vessel into two or more new vessels [1]. The most common pathway for neo-vessel growth in malignancy is angiogenesis (reviewed in [2]) and the process is therefore called tumor angiogenesis.

In 1971, Judah Folkman first advanced the hypothesis that tumor growth depends on angiogenesis [3]. According to this hypothesis, endothelial cells may be switched from a resting state to a rapid growth phase by a diffusible chemical signal emanating from the tumor cells. The switch depends on increased production of one or more positive regulators of angiogenesis, such as vascular endothelial growth factor (VEGF), fibroblast growth factor-2 (FGF-2), interleukin-8 (IL-8), placental growth factor (PlGF), transforming growth factor-beta (TGFbeta), platelet derived growth factor (PDGF), angiopoietins (Angs) and others (reviewed in [4]). These can be exported from tumor cells, mobilized from the extracellular matrix, or released from host cells recruited to the tumor. The switch may also involve down-regulation of endogenous inhibitors of angiogenesis such as endostatin, angiostatin or thrombospondin (reviewed in [5]) and has thus been regarded as the result of tipping the net balance between positive and negative regulators. Mature microRNAs (miRNAs) can furthermore regulate the levels of pro- or anti-angiogenic gene expression at the posttranscriptional level (reviewed in [6]).

Angiogenic signals lead to the preferential differentiation of certain endothelial cells into so-called tip cells, which start to migrate and exist at the leading front of the growing vessels. A number of factors including VEGF receptor (VEGFR)-3 (for lymphatic endothelial cells), VEGFR-1 and–2 (for blood endothelial cells), PDGF-B, and the Notch ligand delta-like ligand (Dll)-4 have been shown to contribute to the endothelial tip cell phenotype [7], [8]. In healthy angiogenesis during development for example, the number of tip-cells are limited leading to an orderly and organized expansion of the vasculature. Endothelial cells located behind the tip cell, so-called stalk cells, express other factors such as VEGFR-1 and Notch-1 and -4 which are important for inducing a quiescent state of these cells [9], [10], maturation of the vascular wall, lumen formation and to support perfusion. However, in pathological angiogenesis including tumor angiogenesis this process is usually disrupted by either excess production of pro-angiogenic signals, lack of angiogenesis inhibitors, path-finding signals or maturation factors, thus leading to excessive tip-cell formation and migration of endothelial cells [11], [12], which do not assume a quiescent phenotype associated with a healthy vasculature.

### 1.1 Structural and dysfunctional features of tumor blood vessels

As a result of the imbalance of angiogenic activators and inhibitors, tumor blood vessels display many structural and functional abnormalities including unusual leakiness (reviewed in [13]), potential for rapid growth and remodeling [14], high tortuosity and sinusoidal appearance (reviewed in [13]), poor coverage by vascular supportive cells including pericytes and smooth muscle cells [15], lack of arterial or venous identity leading to chaotic blood flow, poor functionality and perfusion [16], incorporation of tumor cells into the endothelial wall, alternatively differentiation of tumor stem-like cells to endothelial cells which contribute to the tumor vasculature – a process known as vascular mimicry [17]. These phenotypes, which can be considered “hallmarks of the tumor vasculature”, mediate the dissemination of tumor cells in the bloodstream and maintain the pathological characteristics of the tumor microenvironment.

Tumor vessel density is furthermore very heterogeneous: the highest values are found in the invading tumor edge, where the density is 4–10 times greater than inside the tumor and the arrangement of vessels in the center of a tumor is much more chaotic than at its edges (reviewed in [18]). Importantly, mechanical stress generated by proliferating tumor cells also compress vessels in tumors, with some vessels being oversized, whereas others are more immature and smaller. These structural abnormalities result in disturbed blood flow, hypoxia, hyperpermeability, and elevated interstitial pressure in many solid tumors (reviewed in [19]), responsible, in turn, for impaired delivery of anti-cancer drugs as well as oxygen, the former being critical for the success of chemo- and the latter for radiation therapy.

### 1.2 Tumor hypoxia – an emerging target

There is a complex interrelationship between tumor hypoxia and tumor angiogenesis. The production of several angiogenic cytokines and growth factors is regulated by hypoxia, but as mentioned, tumor angiogenesis also further elevate tumor hypoxia. This vicious circle is critical for driving many of the most pathogenic features of cancer including poor treatment outcome and progression to severe and metastatic disease.

Much of the dynamic regulation of this process involves transcriptionally mediated changes that promote the enhanced production of ligands and receptors, signaling aspects of both mitogenic growth and directing the organization of an endothelial network. While hypoxia and hypoxia signaling play major roles, they are only two means by which angiogenesis can be triggered. Other processes involving proteomic signaling, including those effects directly attributed to the tumor biology [20], can also be activated in support of the angiogenic response to various tissue conditions.

**Fig. 1:**
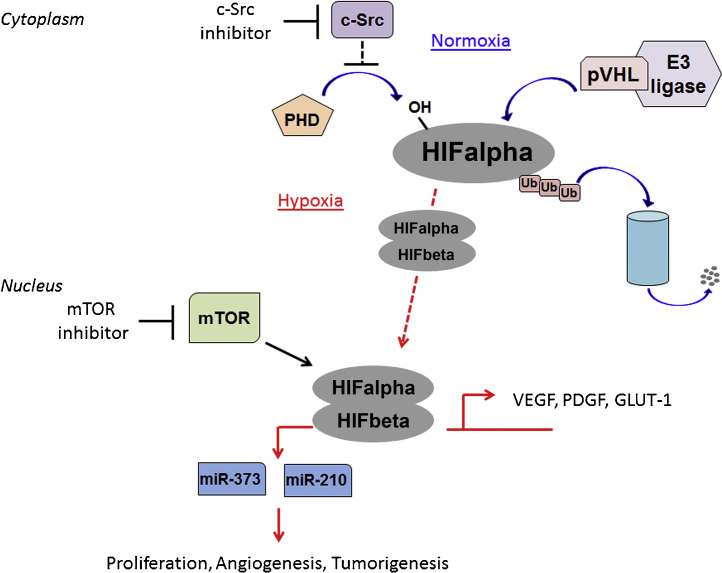
Molecular mechanisms behind HIF regulation and responses in cells. The cellular oxygen sensing response is tightly regulated by a family of prolyl hydroxylases (PHD) which under normal oxygen conditions (normoxia; blue arrows) are responsible for hydroxylating proline residues on hypoxia inducible factor (HIF) alpha subunits. These hydroxylated residues are recognized by a pVHL-E3 ubiquitin ligase complex, whereby HIFalpha subunits are marked for polyubiquitination and subsequent proteosomal degradation. When oxygen levels are low (hypoxia; red arrow) PHDs cannot hydroxylate HIFalphas thereby allowing them to escape pVHL-mediated degradation. HIFalpha subunits accumulate and bind to their heterodimeric partner, HIFbeta, translocate into the nucleus and activate a cascade of hypoxic signaling first by the transcription of various target genes including microRNAs that are important for tumor promoting pathways. Alternatively, c-Src is also capable of activating HIFs by indirectly inhibiting PHD activity via the NADPH oxidase/Rac pathway. mTOR can also promote stabilization and HIF transcriptional activity. Critical points for therapeutic intervention include the use of c-Src and mTOR inhibitors to prevent HIFalpha accumulation and activation.

Hypoxia in tumors develops in the form of chronic hypoxia, resulting from long diffusion distances between (perfused) tumor vessels, and/or of acute hypoxia, resulting from a transient collapse of tumor vessels. One essential pathway activated in this series of events is the activation of hypoxia inducible factors (HIFs), heterodimeric transcription factors composed from alpha and beta subunits, which can be rapidly stabilized to fluidly adapt to and overcome the effects of a hypoxic environment. There are three HIFalpha subunits, HIF1alpha, HIF2alpha, HIF3alpha, which are regulated in an oxygen dependent manner. HIFalpha subunits are hydroxylated by prolyl hydroxylases (PHDs) [21] allowing HIFalpha subunits to be recognized by the von Hippel–Lindau (VHL) ubiquitin-ligase complex [22]. VHL poly-ubiquitinates HIFalpha subunits, leading to their subsequent proteasome-mediated degradation. Under low oxygen conditions PHDs have reduced activity, allowing for HIFalpha subunits to escape VHL-mediated degradation. HIFalpha subunits accumulate in the cytoplasm where they bind HIFbeta to form a heterodimer that subsequently translocates to the nucleus to activate transcription of target genes, including genes important for various processes such as metabolism (glucose transporter (GLUT)-1, hexokinase (HK)-1), cell growth (cyclin (CCN)-D1 [23]) and also angiogenesis, such as erythropoietin, VEGF and PDGF [24] (summarized in Fig. 1). In some cancers, mutations of the machinery (such as VHL or other components) regulating HIF stability can result in oxygen-independent constitutive stabilization of the HIFalpha factors, and as a result these tumors are notoriously highly vascularized [25].

Additional factors besides HIF-mediated VEGF transcriptional activation have also been identified as promoting VEGF expression under hypoxic conditions. Environmental stress as a result of low oxygen and proper nutrient deprivation, such as glucose deprivation, are capable of inducing VEGF mRNA stabilization resulting in increased levels of the secreted ligand and angiogenic growth [26]. Hypoxic stress has also been described as inducing changes in miRNAs which can further influence the host microenvironment with effects on angiogenesis [27].

Targeting hypoxia signaling is a promising approach to provide more options for intervention in this critical pathway of tumor/host biology. Additionally, HIFalpha factors can be regulated by mammalian target of rapamycin (mTOR) family translational signals (Fig. 1), which provides a rich alternate source of targeting (reviewed in [28]), and mTOR drugs are already in the market or emerging.

### 1.3 Tumor lymphangiogenesis and lymphatic metastasis

Metastatic spread of tumor cells, via either blood or lymphatic vascular systems, accounts for the majority of morbidity and mortality in cancer patients. The presence of tumor cells within sentinel lymph nodes (LN) that accept afferent lymphatic vessels draining lymph from the primary tumor often indicates initial metastasis that precedes (or predicts) distant metastasis to other organs. It is also one of the most important markers for predicting patient prognosis and deciding on therapeutic options [29], [30].

Lymphangiogenesis, is often enhanced in malignant tumors, and associated with positive LN metastasis as well as poor survival of cancer patients [31], [32], [33], [34], [35]. Tumor-associated lymphangiogenesis may occur either at the immediate tumor periphery (peri-tumoral lymphatics) or within the tumor mass (intra-tumoral lymphatics), the former having been demonstrated to be functionally responsible for tumor cell dissemination [36], [37]. Tumor lymphangiogenesis is – as tumor blood- (or hem)angiogenesis – also regulated by a balance of pro- and anti-lymphangiogenesis factors. The most frequently studied tumor lymphangiogenic factors are members of the VEGF family, most predominantly VEGF-C and -D, through interactions with in particular VEGFR-3, with some additional evidence for VEGF-A interacting with VEGFR-2 (reviewed in [38]). These factors were found to increase LN metastasis and their expression correlated with poor prognosis in both animal models and human cancers [39], [40], [41], [42], [43], [44]. Other important lymphangiogenic factors include FGF-2 and the key receptor FGF receptor (FGFR)-1 [45], [46], [47], hepatocyte growth factor (HGF) and the cognate receptor c-met [48], insulin-like growth factors (IGF)-1, -2 and IGF receptor (IGFR) [49], [50], EphrinB-2 and Eph receptor tyrosine kinase [51], Ang-1, -2 and Tie2 [52], PDGF-BB and PDGF receptor (PDGFR)alpha and -beta [53], growth hormone and the growth hormone receptor [54], among others.

Tumor cells not only stimulate lymphangiogenesis within or around the primary tumor site, but also have the capability to induce neo-lymphangiogenesis in the LN itself, so as to prepare a “pro-metastatic niche” for the spread of tumor cells [55], [56], [57]. Lymphangiogenesis at the sentinel LN appears to occur beforehand and is further enhanced upon the arrival of metastatic cancer cells [55], [56], suggesting that the LN (possibly conditioned by certain tumor effectors, such as VEGF-A or VEGF-C) helps to provide a favorable environment for tumor metastasis (reviewed in [58]).

As lymphangiogenesis is associated with increased LN metastases (reviewed in [59]), blocking the process (or possibly inducing lymphatic endothelial apoptosis/regression) may serve as a favorable strategy to prevent lymph node metastasis. However, even if the strategy may result in fewer lymph-borne metastases over time, there's still the possibility that other biophysical functions are affected by the alteration in lymphatic flow within and surrounding the tumor. For example, a reduction in lymphatic drainage from the tumor may result in increased interstitial fluid pressure (IFP) within the tumor (reviewed in [60]). This in turn may increase tumor necrosis, hypoxia, and progression, while (at least temporarily) reducing the ability to deliver chemotherapy or other agents via the compressed tumor vasculature.

Adding to the complexity of the regulation and functions of tumor lymphatics, a variety of factors have been found to play key roles in the separation of lymphatic vasculature from blood vessels during development. While these pathways may also contribute to neolymphatic outgrowth in tumors [61], the application of inhibitory strategies to block the relevant pathways/effectors (which include podoplanin, spleen tyrosine kinase (SYK)/SH2-domain containing leukocyte protein of 76 kDa (SLP-76), Rac1/Ras homology gene family member (Rho), and sprout-related EVH1-domain containing (Spred)-1, -2 molecules) and examine the effects on tumor lymphatic investment as well as tumor vascular progression/remodeling has not been examined till date. Whether this might disrupt (or complement the inhibition of) tumor lymphangiogenesis while maintaining the delivery of chemotherapy to tumors during the relevant phase of treatment in the wider cancer treatment program remains to be examined.

### 1.4 Disrupted circadian rhythms in cancer

Social and occupational jetlag is a consequence of the disruption of our internal time-keeping system known as the circadian clock. Social jetlag arise in people who are often rotating between day and night shifts – often seen in healthcare workers, but a feature that is becoming increasingly prevalent in people employed in other types of jobs as well [62]. Occupational jetlag arises from traveling across several time zones and thus exposing oneself to a prolonged, unnatural day/light period, which is often experienced by airline pilots, cabin personnel and globally acting businessmen. It is becoming increasingly clear that such disruptions in the circadian rhythm are associated with higher risk of various diseases, most prominently sleep, metabolic, cardiovascular disorders and cancer [63], [64]. In a large epidemiological study following more than 100,000 American nurses over 10 years, it was found that nurses who worked rotating day and night shifts more than five times per month were at significantly increased risk of various types of cancer [65], [66].

Recently several research groups have found that the circadian rhythm is intimately involved in regulation of angiogenesis both during development [64], [67] as well as in disease [68], [69], [70]. As such, circadian transcription factors were found to directly regulate VEGF levels and were responsible for the elevated night-time spikes in VEGF which are very important for physiological, developmental angiogenesis [64], [71]. Due to the wide-range of cancers that have been associated with disrupted circadian rhythms, and the profound role of angiogenesis in the development of malignancy, it is tempting to speculate that circadian disruption may be an important player in pathological tumor angiogenesis.

## 2 Angiogenesis enables essential tumorigenic pathways

During tumor progression the amount and complexity of deregulated pathways which are essential for full blown malignancy, increase. Whereas pathological deregulation of cell cycle control in (often epithelial) cells is the first step toward tumor development, it is becoming increasingly clear that most of the essential tumorigenic pathways that lead to cancer are dependent on pathological deregulation of non-malignant host cells, and in particular angiogenesis and tumor vascular functions. As such, the tumor vasculature enables pathological tumor metabolism, genetic instability, inflammation, microenvironmental disruption and tumor cell invasion/metastasis.

Tumors are often hypoxic in spite of high vascularization, due to the poor structure and functionality of tumor blood vessels [11], [12]. Intratumoral hypoxia is, somewhat paradoxically, a main cause of high reactive oxygen species (ROS) formation within the tumor cells (reviewed in [72]), and also coupled to pathological tumor cell metabolism and acidosis (reviewed in [73], [74]). Thus, improving the quality of the tumor vasculature has been considered a way to improve perfusion, reduce the pathological leakiness of the tumor vessels and reduce tumor hypoxia [16], [19], which would also result in a more stable tumor genome.

Tumor angiogenesis and pathological activation of the endothelium, tumor vessel leakiness and hypoxia-induced apoptosis/necrosis in the tumor core, are resulting in a massive recruitment and activation of inflammatory cells, such as lymphocytes, neutrophils, macrophages and mast cells. These cells communicate by means of a complex network of intercellular signaling pathways mediated by surface adhesion molecules, cytokines and their receptors. These infiltrating immune cells, generate an environment abundant in growth and angiogenic factors and are implicated in enhancing cancer growth and subsequent resistance to therapy [4], [75]. The inflammatory cytokines interleukin (IL)-1alpha and IL-1beta as well as a wide panel of other signaling molecules produced by infiltrated inflammatory cells including VEGF and matrix-metalloproteinases (MMPs) may contribute to angiogenesis, tumor proliferation, and local invasion of cancer [76], [77].

The deregulated tumor vasculature not only affects the recruitment and activation of inflammatory cells, also cancer-associated fibroblasts (CAFs), myofibroblasts and a number of other cell types, which contribute to tumor progression and resistance to treatment [14], [78], is activated by endothelial cell- or hypoxia-derived factors such as PDGF-BB. On the other hand, CAFs are also rich sources of tumor angiogenic growth factors and cytokines, and thereby play an active role in sustaining tumor angiogenesis and providing resistance to anti-angiogenic therapy.

Tumors may disseminate both by local invasion as well as via blood or lymph vessels (hematologous or lymphatic dissemination). Clinically the latter two processes are the most problematic in most tumor types as they lead to multifocal metastases. Also in tumors that mostly disseminate locally (i.e. neurological cancers for example), this dissemination often occurs via the vasculature as tumor cells coopt the blood vessels and invade the tissue by crawling along the endothelium [79]. Thus in all cases blood and lymphatic vessels in or around the tumor are prerequisite for tumor invasion and metastasis. Metastasis is further enabled due to the poor structural integrity of the tumor blood vessels and pathological angiogenesis-associated tumor hypoxia. Recently, anti-angiogenic therapy has been reported to cause an increased metastatic phenotype, possibly via elevated tumor hypoxia and hypoxia-induced epithelial to mesenchymal transition (EMT) [80], [81], [82]. Anti-angiogenic therapy may however also increase the metastatic potential of tumor cells through adaptive resistance pathways not associated with hypoxia [83], indicating that anti-angiogenic therapy-induced changes in the tumor phenotype may lead to a more aggressive disease through a number of different mechanisms. It is therefore not clear if targeting the tumor vessels would be beneficial or detrimental from a tumor metastasis point of view. However, it may be possible to merely reduce tumor vascularization, improve the structure of the tumor blood vessels and perfusion in the tumor and thus reduce the pathological characteristics of the vessels, for example via sub-maximal dosing of anti-angiogenic drugs [19]. There are still not much clinical data supporting a beneficial role for promoting formation of less pathological vessels in tumors, and it is not known how to best achieve this in patients.

## 3 Targets for anti-angiogenic therapy

The complexity in the angiogenic system provides many targets for therapeutic intervention. On the other hand redundancy in the angiogenic pathways raises the possibility of resistance to selective therapeutic agents (reviewed in [84], [85]). Examples of agents that target circulating angiogenic factors include monoclonal antibodies targeted against VEGF (bevacizumab) [86] or fusion proteins that trap angiogenic factors (aflibercept or AMG386) [87]. Agents that target synthesis of angiogenic factors include inhibitors of mTOR, cyclo-oxygenase (COX) or heat shock protein 90 (HSP90) [88], [89]. These groups of agents in addition to inhibiting the synthesis of angiogenic factors can inhibit several other aspects of cancer biology such as growth, resistance to apoptosis or metastasis. Agents that target the angiogenic receptors are mainly tyrosine kinase inhibitors (sorafenib, sunitinib, pazopanib, regorafenib or axitinib) with multiple targets [90], [91], [92]. These agents are currently being used in the treatment of several malignant diseases ranging from breast, lung, gastric, colorectal, hepatocellular, glioblastoma, and neuroendocrine tumors.

Similarly, there are several agents in clinical trials aimed at blocking lymphangiogenesis/metastasis, mostly via neutralizing VEGF-A, -C or -D-induced receptor activation. For example, a major approach involves application of a variety of tyrosine kinase inhibitors such as Ki23057, used to block gastric cancer spread in mice through blockade of VEGFR3 autophosphorylation [93]. Other agents in clinical testing include PTK787/ZK222584 (Phase III – colorectal cancer); BAY43-9006 (Phase II, multiple carcinomas); CEP7055 (Phase I – various malignancies); or JNJ-26483327 (Phase I for multiple advanced solid tumors). These, among other anti-lymphangiogenic agents under study are reviewed in Ref. [59].

It is worthwhile mentioning that hormone- and chemotherapy could also have anti-angiogenic activity, particularly metronomic therapy which refers to the frequent, even daily, administration of chemotherapy (e.g. cyclophosphamide, methotrexate or capecitabine) in doses below the maximum tolerated dose, for long periods of time, with no prolonged drug-free breaks [94], [95]. Other chemotherapeutics, routinely used in clinic, may also have anti-angiogenic activity in vitro or in vivo [96] as: (1) paclitaxel [97], doxorubicin and thalidomide [98] which seems to be mediated via inhibition of VEGF and bFGF [99]; (2) celecoxib, which may cause a time-dependent reduction in circulating angiogenic markers; (3) bisphosphonates may have anti-angiogenic effects [100] via reduction of VEGF and PDGF serum levels [101]; (4) PI3K inhibitors (including rapamycin analogues as temsirolimus (CCI-779) and everolimus (RAD001)) decrease tumor angiogenesis [102], [103], [104] via the inhibition of HIF-1alpha caused by the blockade of mTOR activity.

Trials that have combined monoclonal antibodies and tyrosine kinase inhibitors have given rise to an increase in the side effects profile. A more rational approach would be to consider combinations of agents that block production of angiogenic factors with such that target angiogenic factors or receptors. The rationale behind such combinations include the fact that anti-angiogenic agents can improve the delivery of cytotoxic agents to the tumor site, may alter hypoxia in the tumor and sensitize it to chemotherapy or may impede the ability of the tumor to recover from cytotoxic effects of chemotherapeutic agents [105]. As tumors express more than one angiogenic cytokine and the fact that during tumor progression the palette of tumor-derived angiogenic factors grows more and more complex, any single inhibitor would not be sufficient for achieving sustained anti-tumor responses [74]. We hypothesize that simultaneously hitting multiple important aspects of tumor angiogenesis, each outlined in detail in the sections above, with a cocktail of compounds might create a more effective treatment. This could particularly be the case for indications such as cancer prevention in high risk settings, or maintenance therapies.

To facilitate the use of plant-derived compounds in cancer treatment, we have selected 10 key mechanisms that lead to pathological growth and functions of the tumor vasculature, such as EC migration/tip cell formation, phenotypic changes in the tumor microenvironment or pathogenic activation of stromal cells (macrophages or fibroblasts). These pathways (top row in Table 1) were selected as candidate targets for anti-angiogenic natural compound-based therapy development.

## 4 Therapeutic potential of plant-derived compounds

During the last decades, phytochemicals have gained significant recognition for their potential therapeutic uses in cancer [106], [107], [108]. Extensive research has revealed enormous potential and exciting pharmacological properties of plant-based medicinal compounds, and demonstrated synergistic effects in combination with other agents to inhibit tumor angiogenesis, although the use of phytochemicals alone is still a limited option for cancer treatment. Some phytochemicals used in cancer therapies demonstrate relatively low side-effects, and some even limit the side-effects of chemotherapeutics or anti-angiogenic drugs. Fruits, vegetables, cereals, pulses, legumes, herbs, spices and medicinal plants – such as *Artemisia annua* (Chinese wormwood), *Viscum album* (European mistletoe), *Curcuma longa* (turmeric), *Scutellaria baicalensis* (Chinese skullcap), *Vitis vinifera* (grape seed extract), *Magnolia officinalis* (Chinese magnolia tree), *Camellia sinensis* (green tea), *Ginkgo biloba* (ginkgo), *Poria cocos* (tuckahoe), *Zingiber officinalis* (ginger), *Panax ginseng* (ginseng), *Rabdosia rubescens hora* (rabdosia), and Chinese destagnation herbs – are all considered to be good sources of phytochemicals exhibiting anti-cancer, and in particular anti-angiogenesis activities. The active ingredients in these plants are sometimes extracted and given in doses higher than what can be achieved from consuming the plants of which they are derived in order to give stronger therapeutic effect.

Many medicinal herbs and purified phytochemicals have recently been evaluated for anti-lymphangiogenic and anti-angiogenic properties in cancer (reviewed in [109], [110], [111]). The potential mechanisms underlying their anti-lymphangiogenic features involve (1) the control on cell proliferation, tube formation and cell cycle progression of lymphatic endothelial cells, as exhibited by multiple compounds fractionated from Korean and Japanese *Saussureae radix*, *Psoraleae semen* and *Aurantti fructus immaturus* [112]; (2) the inhibition of COX-2 expression and IL-1beta production and the subsequent reduction in VEGF-C-induced VEGFR-3 phosphorylation, as observed with wogonin [113]; and (3) the down-regulation of VEGFR3 and small GTPases, as well as the inhibition of VEGFR3-mediated extracellular-signal regulated kinase (ERK)-1 and -2 phosphorylation by cryptotanshinone [114]. The mechanisms behind their anti-angiogenic effects include: (1) inhibition of MMPs, (2) prevention of capillary sprout formation, endothelial cell proliferation and migration, and (3) modulation of angiogenic ligand/receptor mediated signaling pathways.

MMP inhibition blocks the degradation of endothelial basement membrane proteins, a process that otherwise leads to increased permeability and poor structure and stability of the tumor vasculature as well as release of sequestered angiogenic factors. However, caution is warranted regarding the effects on MMP/tissue inhibitor of metalloproteinases (TIMP) system as MMP inhibitors in clinical trials have failed and even induced worse survival rates compared to placebo treated patients [115]. In addition, several experimental studies have shown potent anti-tumorigenic activities of many MMPs including MMP-9 [116], [117], [118], [119], [120], [121]. In the case of TIMPs both tumor protective and tumor enhancing properties have been reported [122], [123], [124], [125]. For example, in breast cancer patients, high tumor and serum levels of TIMP-1 have been associated with decreased response to chemotherapy and consequently reduced survival [126], [127], [128].

Targeting capillary sprout formation and directional endothelial cell migration may depend on diverse inhibitors of the Notch signaling pathway, for which commercial drugs are in development. There are also phytochemicals which exhibit such effects.

Finally, angiogenic signaling inhibitors include the modulation of mitogen-activated protein kinase (MAPK) and Akt signaling, inhibition of activator protein (AP)-1 activation, and the down-regulation of VEGF, transforming growth factor (TGF)-beta, MMP-9, as well as the upregulation of TIMP-1 which lead to reduction of tumor cell invasion and blood vessel growth [129], [130], [131], [132].

### 4.1 Tea polyphenols

Tea is one of the most highly consumed beverages in the world and is rich in compounds exhibiting multiple health benefits (reviewed in [108]). The pharmacological action of tea is mainly attributed to large quantities of polyphenolic compounds known as catechins, which include epicatechin (EC), epigallocatechin (EGC), epicatechin-3-gallate (ECG), and epigallocatechin-3-gallate (EGCG) particularly in green tea and thearubigin and theaflavins in black tea. Tea polyphenols have been shown to act on angiogenesis through different signaling pathways. For instance, EGCG has been found to directly inhibit capillary endothelial cell proliferation at low concentrations, which illustrates the importance of this molecule as an important tumor angiogenesis inhibitor [133]. EGCG also inhibits VEGF production in MDA-MB231 breast cancer cells and human umbilical vein endothelial cells which correlate with the inhibition of protein kinase C, c-fos and c-jun RNA transcripts, suggesting that AP-1 responsive regions present in the human VEGF promoter may be involved [134]. In neuroblastoma, fibrosarcoma, glioblastoma, prostate cancer, and human gastric cancer cells, EGCG inhibited MMP-2 and MMP-9 while inducing the activity of their inhibitors TIMP-1 and TIMP-2 [135], [136], [137], [138]. In human breast cancer cells, EGCG treatment reduced MMP-2 activity and the expression of focal adhesion kinase (FAK), membrane type-1-MMP (MT1-MMP), nuclear factor (NF)-kappaB, VEGF, and the adhesion of cells to the extracellular matrix (ECM) [139]. Similar signaling pathways have also been demonstrated in animal studies. EGCG also targets urokinase plasminogen activator (u-PA), leading to a down-regulation of VEGF production in tumor cells and subsequent repression of AP-1, NF-kappaB and signal transducer and activator of transcription (STAT)-1 transcription factor pathway [140], [141]. EGCG furthermore inhibits aryl hydrocarbon receptor (AhR)-AhR-mediated transcription by binding to HSP90. Furthermore, a range of semi-synthetic and synthetic derivatives are more potent than EGCG in a luciferase refolding assay for HSP90 activity [106]. In transgenic adenocarcinoma of the mouse prostate (TRAMP) mice, green tea polyphenol infusion resulted in marked inhibition of effectors of angiogenesis and metastasis, notably VEGF, uPA, MMP-2, and MMP-9 [142]. In a dimethylaminoazobenzene (DAB) induced hepatoma model, administration of black tea polyphenols not only reduced the incidence of invasion, but also inhibited tumor hypoxia and angiogenesis [143]. In addition, EGCG targets tissue plasminogen activator (t-PA), which is one of the critical proteases that enable tumors to metastasize [144]. Several studies based on cell culture and animal models further demonstrated the cancer preventive function of tea, which is associated not only with the inhibition of VEGF, NF-kappaB, c-fos and cyclin D1 promoter activity, but also the decrease of Bcl-XL and the stabilization of p53 [145], [146].

### 4.2 Curcumin

Curcumin is a polyphenol isolated from *Curcuma longa*. As a yellow dye, curcumin has been used widely for centuries not only in cooking, but also in traditional therapies for various diseases, especially inflammatory diseases. Curcumin and tetrahydro-curcumin, one of its metabolites have been extensively investigated as anti-inflammatory and anti-cancer molecules [147], [148]. Curcumin inhibits the expression of epidermal growth factor receptor (EGFR), VEGFR-1, VEGFR-2 and VEGFR-3, and the kinase activity of Src and FAK, which are responsible for the induction of angiogenic genes as well as endothelial cell polarity and migration [149]. Curcumin also reduces the MMP-2 and MMP-9 expression, along with the suppression of growth and invasion potential of tumor cells in culture and xenograft experiments [150]. Oral administration of curcumin in nude mice xenografted with hepatocarcinoma cells led to significant lowering of tumor neocapillary density. The expression of angiogenic biomarkers COX-2 and serum levels of VEGF were significantly reduced in the curcumin-treated group [151]. This inhibition of both VEGF and VEGFR in different cancers, has fueled the interest in this compound for further investigations as a potential anti-angiogenesis agent [152].

### 4.3 Resveratrol

Resveratrol (3,4′,5-trihydroxy-trans-stilbene), a dietary polyphenol derived from grapes, berries, peanuts and other plant sources has been shown to affect several steps in the tumorigenic process including tumor angiogenesis [153], [154], [155]. Resveratrol inhibits capillary endothelial cell growth and new blood vessel growth in animals [130]. It also prevents diethylnitrosamine-initiated and phenobarbital-promoted hepatocarcinogenesis in rats through interrupting cell proliferation, inducing apoptosis [155] and impeding angiogenesis by suppressing VEGF expression through down-regulation of HIF-1alpha [156]. In in vitro studies, resveratrol was reported to inhibit cell proliferation of human ovarian cancer cells and human osteosarcoma cells by attenuating HIF-1alpha [157], [158]. Through abrogation of VEGF-mediated tyrosine phosphorylation of vascular endothelial cadherins and beta-catenin, resveratrol further prevents cytokine-induced vascular leakage and tumor metastasis [130]. The underlying molecular mechanisms include: blocking VEGF- and FGF-receptor-mediated MAPK activation, inhibiting Akt- and MAPK-driven HIF-1alpha basal expression and its induction by IGF-1, stimulating the proteasomal degradation of HIF-1alpha, inhibiting phosphatidyl inositol (PI)-3K/Akt and Ras/mitogen/extracellular signal-regulated kinase (MEK)/ERK pathways, and activation of forkhead box (FOX)O transcription factors [130], [157], [158], [159].

Furthermore, the synthetic stilbene derivatives of resveratrol have stronger inhibitory effect on angiogenesis than resveratrol, as measured by cell proliferation and tube formation in bovine aorta endothelial cells [160]. For instance, the stilbene derivative rhapoantigenin inhibits angiogenesis-induction by prostate cancer cells through HIF-1alpha degradation [161]. Likewise, resveratrol and a series of natural or synthetic stilbenes inhibit the growth of colon cancer-xenografts in mice through attenuation of VEGF and pro-MMP-9 [162]. While pre-clinical studies have been done broadly and for many different indications, resveratrol is yet to be clinically assessed for safety and prevention of cancer as well as in cancer therapeutic regimens.

### 4.4 Flavonoids

In the family of polyphenols, flavonoids have been found to suppress tumorigenesis via anti-angiogenesis, anti-oxidant as well as anti-proliferation effects on tumor- as well as tumor-associated stromal cells including endothelial cells [163], [164]. Flavonoids, including flavones (apigenin, luteolin), flavanols (quercetin, kaempferol), flavanones (hesperetin, naringenin), anthocyanins (cyanidin, delphinidin) and isoflavones (geneistein, daidzein) function as scavengers of free radicals and thus inhibit ROS formation and hypoxia-signaling cascades, modulate COX-2, and inhibit EGFR, IGFR-1 and NF-kappaB signaling pathways [165]. For instance, kaempferol (3,5,7-trihydroxy-2-(4-hydroxyphenyl)-4H-1-benzopyran-4-one) has been reported to reduce ovarian cancer risk [166], [167]. Kaempferol exhibits anti-inflammatory effects through inhibition of IL-4 [168] and COX-2 expression by suppressing Src kinase [169], and further down-regulates the NF-kappaB pathway [170]. Flavonoids furthermore inhibit angiogenesis through multiple mechanisms, including interaction with the COX-2 and lipoxygenase-5 enzymes, EGFR and the human epidermal growth factor receptor (HER)-2 intracellular signaling pathway [171].

Silymarin is the standardized extract of the seeds of *Silibum marianum* (milk thistle). Silibinin, the major active constituent was initially developed as a hepatoprotective product. Recently, however, it has been reported that silibinin inhibit MMP-2 expression and suppresses capillary formation of human umbilical cord venous endothelial cells (HUVECs) on matrigel [172], [173], [174]. Other naturally occurring flavonoids have been showing anti-angiogenesis and anti-oxidant effects via interference with diverse signaling pathways. For example, myricetin has been shown to inhibit inhibitor of kappaB kinase (IKK) kinase activity and prevent degradation of I-kappaBalpha and I-kappakBbeta in tumor necrosis factor (TNF)-activated endothelial cells [175]. Sulforaphane has been demonstrated to inhibit VEGFR2 at the transcriptional level [176].

Studies also showed that licochalcone A (LicA), a major constituent of *Glycyrrhiza inflata*, significantly inhibits proliferation, migration, and tube formation of HUVECs as well as microvessel growth from rat aortic rings. Furthermore, LicA significantly inhibits the growth of CT-26 colon cancer implants in BALB/c mice, with fewer CD31- and Ki-67-positive cells but more apoptotic cells [177]. Isoliquiritigenin, another flavonoid found in *G. inflata* inhibits cell migration and invasion of human prostate cancer cells (DU145 and LNCaP) mediated by decreased c-Jun N-terminal kinase (JNK)/AP-1 signaling and reduced production of proangiogenic factors [178]. Taken together, these findings provide evidence that flavonoids inhibit angiogenesis in vitro and in vivo, via antioxidant, anti-inflammatory and anti-angiogenic signaling pathways.

### 4.5 Terpenoids

Terpenoids are the most diverse constituents in many plant species. They form a group of natural substances which includes steroids and sterols, exhibiting anti-inflammatory and anti-carcinogenic properties [20], [179], [180], [181], [182].

The bioactive terpenoid, tripterine, also known as celastrol, a quinine methide triterpenoid is the most abundant bioactive compound derived from the root of *Trypterigium wilfordii*. Tripterine modulates the expression of proteins with angiogenic and metastatic activities (MMP-9, intercellular adhesion molecule (ICAM)-1, VEGF) and those involved in cell survival (inhibitor of apoptosis protein (IAP)-1, X-linked (X)-IAP, B-cell lymphoma (Bcl)-2, Bcl-xL, flice inhibitory protein (cFLIP), survivin) or cell proliferation (cyclin D1, COX-2) in tumor cells [183], [184]. These findings provide evidence for its potential anti-angiogenic and anti-tumor activities.

Escin is a pentacyclic triterpenoid which is isolated from the seeds of *Aesculus hippocastanum* (horse chestnuts) [185]. Escin sodium has been shown to inhibit endothelial cell migration and motility. This anti-angiogenic activity was mediated partly by inhibiting ERK and p38 MAPK pathways, which are involved in cell proliferation, motility and apoptosis [186].

Withaferin A is a major steroidal lactone constituent of the medicinal plant *Withania somnifera*, consumed as a dietary supplement around the world and used in the treatment of tumors and inflammation in several Asian countries [187]. Withaferin A exerts potent anti-angiogenic activity in vivo even at 500-times lower dose than that exerting direct anti-tumor activity [188], [189]. Similar trends toward a more potent anti-angiogenic rather than anti-tumorigenic effects are also observed for other phytochemicals (such as tubulysin A as discussed below) suggesting that the vasculature may be a good target for treatments with phytochemicals.

In addition, carotenoids have anti-cancer activity in breast cancer animal models. The carotenoid group includes alpha-carotene, beta-carotene, lycopene, lutein, astaxanthin, cryptoxanthin and zeaxanthin [190], [191]. The anti-oxidant action is one of the presumed mechanisms for the anti-angiogenic effects of the carotenoids.

### 4.6 Phytoestrogens

Phytoestrogens are plant compounds which are structurally similar to estrogen. Thereby, these compounds may have beneficial effects in prevention of steroid hormone-dependent cancers such as breast and prostate cancer. There are two major classes of phytoestrogens, isoflavones, found in soy at high concentrations, and lignans, found in flaxseed in high amounts. Lignans are ubiquitous compounds in plant material and are present in seeds, whole grains, vegetables, and berries [192], [193], [194]. Genistein (GEN), the representative of isoflavone, and enterolactone (ENL) is the main active and most potent metabolite of lignin, both have agonistic and antagonistic actions in cancer, depending on their dose and the tumor-type. In Eastern societies, the diet contains large amounts of phytoestrogens, and accordingly, the incidence of cancers such as breast cancer and prostate cancer is low [195], [196]. In the diet, phytoestrogens are consumed in combination and various phytoestrogens may have different effects on tumor biology. GEN has been shown to reduce the angiogenic and metastatic potential of various cancer cell types in vitro [197]. However, the pro-estrogenic effects of GEN in breast cancer [198] suggest that caution with this particular phytoestrogen in hormone-dependent cancers is warranted. In addition, as the major circulating lignan in the human body, the majority of dietary lignans are converted into ENL. ENL inhibits cancer growth in vivo by enhancing apoptosis, inhibiting angiogenesis, and reducing inflammation without severe side-effects [199], [200], [201].

## 5 Prophylaxis, pharmacodynamics and safety issues

As outlined above, there is convincing evidence in support of beneficial effects of phytochemicals in cancer-related pathways, particularly with regard to anti-angiogenesis. Furthermore, many natural molecules exhibit potent anti-angiogenic activity similar to the synthetically produced drugs currently in clinical use. For example curcumin, EGCG, finasteride and barrigtozenol demonstrated comparable effects on VEGF receptor inactivation as pazopanib, the reference drug [202]. 10 μM curcumin significantly inhibited VEGF-induced HUVEC migration to a higher degree (52%) than the same concentration of a synthetic anti-angiogenic agent – the selective PDE4 inhibitor rolipram (41%) [203]. Furthermore, the EC50 dose–responses are usually lower for anti-angiogenic effects vs. cytotoxic effects on cancer cells both for synthetic and natural products. For example tubulysin A, a natural compound of myxobacterial origin, which inhibits tubulin polymerization has an EC50 for endothelial cell proliferation of 1.2 nM and up to 3.0 nM for cancer cell proliferation [204], [205]. The synthetic derivates of tubulysin A, AU816 and JB337, exhibit similar differences; EC50 of AU816 for endothelial cell proliferation is 4.4 nM, whereas inhibition of cancer cell proliferation is 10 nM and for JB337 the EC50 levels are 14 nM compared to 100 nM for endothelial and cancer cell proliferation, respectively [204], [206].

Since most phytochemicals have poor water solubility and low bioavailability, the use of nano-carriers allows the preparation of these compounds in solid or liquid formulations with highly improved pharmacodynamics properties. Such encapsulation, however, render these natural compounds semi-synthetic, but the active ingredients in such preparations are still all-natural. Often, delivery mechanisms based on nano-carriers may in addition to improved pharmacokinetics also lead to improve targeting of the active compounds to the appropriate – i.e. tumor – site. For example in the case of anti-lymphatic drugs, nano-carriers, which are not able to cross the endothelium by themselves, but extravasate in tumors due to the high leakiness of tumor blood vessels, accumulate in draining intra-tumoral lymphatics and thus serve as a means to target the drugs to the leaky tumor environment. Nanoparticles made of biodegradable polymers have been utilized recently for the delivery of anti-cancer phytochemicals [207], [208]. Siddiqui et al. [209] reported that nano-encapsulated EGCG retains its biological effectiveness, with over 10-fold dose advantage compared to nonencapsulated EGCG for exerting its pro-apoptotic, and anti-angiogenic effects. Encapsulation of kaempferol with nanoparticles significantly reduces cancer cell viability, as does coating it onto poly(dl-lactic acid-coglycolic acid) (PLGA) nanoparticles [210]. Furthermore, nano-encapsulated curcumin has increased in vivo tumor anti-angiogenic activity relative to its non-encapsulated form [211]. Solid lipid nano-encapsulation of berbarine also increased inhibition of cancer cell proliferation, cell cycle arrest, and apoptosis [212]. Nano-encapsulation therefore seems like a promising strategy to overcome pharmacodynamics issues associated with many phytochemicals.

The bioavailability, efficacy and stability of phytochemicals may also be increased by synthetic derivation – a procedure in which functional groups are added to or removed from the natural phytochemical, leading to improved pharmacokinetics of the resulting semi-synthetic phytochemical drug, while retaining or even improving its biological effects. Such an approach has proven particularly useful in rendering oleanolic acid attractive for therapeutic applications. Oleanolic acid is an oleanane-type triterpenoid that is widely found in dietary and medicinal plants [213]. Studies demonstrated that oleanolic acid has anti-angiogenic effects on bovine aortic endothelial cells and in the chick embryo chorioallantoic membrane assay [214]. However, 2-cyano-3,12-dioxoolean-1,9-dien-28-oic acid (CDDO), the synthetic derivative of oleanolic acid, its C-28 methyl ester (CDDO-Me) and C-28 imidazole (CDDO-Im), are all shown to be potent inhibitors of angiogenesis in the Matrigel sponge assay and in tumors established from immortalized Kaposi's sarcoma cells. These compounds prevent endothelial cell tubulogenesis on Matrigel and inhibit the VEGF-induced ERK-1 and -2 pathway in HUVECs [214], [215]. It has been suggested that COX-2 overexpression may lead to increased tumor angiogenesis [216], [217]. Therefore, inhibition of the COX-2 pathway might prevent carcinogenesis and angiogenesis via anti-inflammatory signaling. For example, CDDO-Me has been shown as an effective inhibitor of COX-2 and tumor growth in breast cancer models in mice.

These studies underscore the importance of phytochemicals and semi-synthetic derivatives or formulations of phytochemicals, as a novel anti-tumor treatment option in the future. Moreover, their anti-angiogenic properties could help to optimize the effectiveness of existing chemotherapeutic drugs. The use of phytochemicals as adjuvants in combination with anti-angiogenic drugs is promising. For example, tumor hypoxia can be a deleterious result of anti-angiogenic cancer treatment leading to changes in cellular metabolism [73], [74], [218], elevated intracellular ROS-levels [72], de-differentiation of tumor cells and increased metastatic propensity [218], [219]. These hypoxia-induced pathways can be treated by various phytochemical antioxidants including EGCG, melatonin, resveratrol and silibinin [143], [157], [220], [221], [222], [223]. While this could provide a strong argument for synergy by combining such agents, it was traditionally believed that hypoxia should be the very goal of anti-angiogenic treatment and an important inducer of tumor cell apoptosis [224], [225]. Therefore addition of such antioxidants may reduce the hypoxia-induced cell death, as it does for non-malignant cells [226], [227], potentially leading to a poorer outcome from the anti-angiogenic treatment. Also in the interaction between tumor inflammation and angiogenesis the combination of anti-inflammatory phytochemicals with anti-angiogenic drugs may provide a potential for synergy as inflammatory cells are considered a source of alternative angiogenic factors and thus important for evasive resistance [4]. However, some inflammatory cells are thought to be anti-tumorigenic and important for tumor clearance [228], [229], and inhibiting them could lead to antagonistic interactions in such combinations. Thus each combination of phytochemicals and other treatment options should be thoroughly tested in pre-clinical and clinical trials before adopted by medical practitioners.

Tumor angiogenesis is not only required for late-stage progressive disease, but is crucial even for initial growth of the tumor to clinically detectable masses. Therefore, the inhibition of angiogenesis has been proposed as an important preventive strategy [230]. This idea, also known as angioprevention, is highly efficacious in preventing tumor growth in animal models [156], [231]. Angioprevention should be achieved using non-toxic agents such as phytochemicals, as they are often consumed on a daily basis. Ideally, we should however also aim to achieve anti-angiogenic treatment efficacy using phytochemical agents at sufficiently low doses to keep toxic side-effects are kept to a minimum. Thus safety concerns should weigh in heavily when deciding on the most appropriate drugs for anti-angiogenic treatment in cancer. Here, we intend to identify 10 suitable, prototypical anti-angiogenic therapeutic approaches based on phytochemicals that have proven to be effective against our previously defined targets. We aimed at finding compounds that are free from intellectual property constraints, readily available and cheap such that everyone, all over the world, could use them as an alternative or complementary treatment option. A modern approach to chemotherapy may be to use lower doses of combinations of chemicals, which work in complementary ways. Thus, while low-doses of a single compound may not achieve the full anti-angiogenic function, a mixture of compounds, or extracts from medical plants, may be able to specifically synergize in preventing angiogenesis while avoiding significant side-effects. Such an approach, combining phytochemicals in tea and soy extracts has been successfully applied to the anti-angiogenic treatment of prostate and breast cancer in animal models [47], [49]. Therefore we aimed at selecting phytochemicals from as many different classes of natural compounds listed in Section 4 as possible.

In line with this rationale, we have identified 10 natural compounds as potential therapeutic approaches (top row of Table 2) against the previously identified targets (top row of Table 1). These compounds are safer than the synthetic anti-angiogenesis inhibitors in clinical use today [232], [233]–and may even be considered as prophylactic (angioprevention) agents. These molecules were selected because they exhibit pleiotropic functions, i.e. disrupting both inflammation and vascularization during tumor onset and progression and while some of them share molecular targets, combined they will inhibit a broad spectrum of pro-angiogenic signaling pathways lowering the risk of adaptive resistance. As tumors often progressively turn on pro-angiogenic pathways during their development to more advanced stages (reviewed in [234]), which is one possible reason why many patient do not respond to single-agent therapies such as anti-VEGF drugs, the cocktail of 10 phytochemical-based therapeutic approaches mentioned here would be a more broad-acting treatment option, not least for such patients that do not respond to commercially available anti-VEGF drugs.

## 6 Validation of targets and approaches

Anti-angiogenic agents have demonstrated activity in a broad spectrum of malignancies. The questions of finding the best drug, or combination of drugs, for each patient and how to best evaluate the response to treatment are still not adequately answered. An alternative to current efforts on targeted therapy development could be to inhibit an array of pathological targets of tumor blood vessels such as the 10 targets identified here (Table 1, top row). We envision that such a broad approach, simultaneously hitting multiple or all of these targets of the tumor vasculature at the same time, would produce an improved therapeutic outcome compared to single-target therapy. The current strategy we endorse for further investigation considers a combination of phytochemicals, identified to act on more than one or a few of these targets (Table 2, top row). We believe that this approach circumvents the numerous avenues of emergent resistance, and has the potential to be an active therapeutic in a broader range of tumors. We encourage researchers and clinicians alike to test formulations based on this natural compound cocktail-principle and in this way further establish the paradigm of phytochemicals as anti-angiogenic treatment options in cancer.

Given the extensive cross-talk between mechanisms regulating angiogenesis and other aspects of cell biology as well as the broad effects of phytochemicals, we believe that it is important to be able to anticipate synergies that might be achieved through acting not only on aspects of tumor angiogenesis but also on other essential aspects of tumor biology such as the “hallmarks” of cancer. Accordingly, the identified targets for anti-angiogenic therapy and the therapeutic phytochemical-based approaches were cross-validated against their effects on other “hallmark” processes, by undertaking a thorough, qualitative background literature research. An independent team of scientists consisting of specialists in various “hallmark” areas specifically sought to determine the relevance of our identified targets and the nominated phytochemical approaches across these other important areas of cancer biology. The result of this elaborate cross-validation effort is shown in Table 1, Table 2, for the targets and suggested phytochemical-based approaches respectively. We found that many of our identified targets and approaches were also relevant for other aspects of cancer biology. In such cases they were noted as having “complementary” effects (denoted with a + in Table 1, Table 2). In instances where reports on relevant actions in other aspects of cancer biology were mixed (i.e. reports showing both pro- and anti-potential), the effects are considered “controversial” and denoted +/− in the tables. Finally, in instances where no literature was found to support the relevance of a target or approach in other aspects of cancer biology, we consider that there is “no known relationship” and denote this with a 0 in the tables. We did not find any instances in which our targets or approaches acted to promote other pathological aspects of tumor biology. As an example, reducing tumor hypoxia would also lead to reduced tumor-promoting inflammation and therefore we mark this with a + in the cell intersecting the “(reduce) hypoxia” column with the “tumor promoting inflammation” row in Table 1. Conversely, our suggested approach resveratrol was found to both inhibit and induce metastasis and we therefore denote this as +/− in the cell intersecting the “resveratrol” column with the “Invasion and metastasis” row in Table 2.

As it can be determined from closely inspecting these tables, most of our targets and approaches are found to also inhibit other essential aspects or hallmarks of tumor pathology. As such, we believe that the use of the suggested phytochemical approaches – or similarly acting derivatives or analogues – would be effective as alternative anti-angiogenic agents while also inhibiting multiple other aspects of tumor pathology at the same time. Especially oleanolic acid, silibinin, curcumin, EGCG, melatonin and resveratrol, have strong synergistic actions, i.e. anti-tumorigenic effects in almost all other hallmark areas in addition to being effective anti-tumor-angiogenic agents. It would be highly important to experimentally test the effects of such a cocktail of phytochemicals in pre-clinical and clinical studies in the future.

## 7 Future directions

The VEGF pathway holds the key in regulating angiogenesis and targeted therapies against this pathway have been established in the clinic. However, after initial positive results several studies have failed to show increased survival rates for many groups of patients treated with anti-VEGF drugs. It is clear that multiple factors contribute to enabling the angiogenic switch in tumors. However, till date an overwhelming focus has been placed on blocking the VEGF-VEGFR2 pathway. Using small synthetic molecules with a broader target profile, it has been possible to achieve better anti-angiogenic effects in tumors compared to using specific antibody based therapeutics. However, the former are more toxic, still not sufficiently effective and therefore other strategies need to be considered.

It is becoming increasingly clear that plant-derived medicinal compounds may in many cases be at least as effective in blocking angiogenesis as the currently used synthetic drugs, while exhibiting only a fraction of the negative side-effects. Some of these compounds may, however, be more effective in treatment of some tumors, such as the potential for using phytoestrogens in treatment of ER-positive breast cancer or that of melatonin in treating cancer in people with disrupted circadian rhythms. Identification of suitable biomarkers for selection of patients to different types of therapeutic regimens as well as response to treatment has however so far not been achieved but remain a very important avenue for future research.

The desired optimal effect of anti-angiogenic treatment is another issue, which requires further study. Is complete vascular regression what we aim for? Such an outcome would be expected to result in extensive tumor hypoxia, radically reduced perfusion and thus reduced delivery of not only oxygen and nutrients but also drugs to the tumor tissue. Some studies argue that such a result of anti-angiogenic treatment may in fact lead to a more invasive tumor phenotype and worse prognosis compared to the non-anti-angiogenesis treated group. Perhaps it would be a better option to merely reduce the vascular density, improve the structure and function of tumor blood vessels such that they lose their pathogenic characteristics and more closely resemble blood vessels in healthy tissues. Such a phenotype would be expected, however, to lead to improved perfusion, oxygenation and delivery of nutrients to the tumor, thereby potentially increasing tumor growth rate. On the other hand, this would improve the effects of co-administered chemotherapy, which would also be delivered more effectively to the tumor, and which would more effectively kill the tumor cells if these were well nourished and actively proliferating. Perhaps it should even be considered to withdraw anti-angiogenic treatment if it is found to result in dramatically reduced perfusion and oxygenation of the tumor, at least if such treatment is used in combination with chemo- or radiotherapy. The phytochemical approaches to inhibit tumor angiogenesis identified in Table 2 can, however, in addition to pruning non-perfused and pathological blood vessels in the tumor, also reduce the detrimental effects of chemotherapy, leading to improved tolerance and therefore improved health and potentially extended survival when used in combination with classical cytostatic therapies. It should therefore be considered whether such compounds could be beneficial for tumor patients receiving chemotherapy, even if they are also receiving anti-VEGF drugs as the ones in clinical use today do not reduce the side-effects associated with chemotherapy.

While many natural compounds have general beneficial effects, it is clear that some compounds are more effective in cancer treatment than others. For example, a recent meta-analysis of randomized placebo-controlled trials testing the effect of dietary supplementation of multi-vitamins and multi-minerals, failed to show any effect on cancer mortality suggesting that this is not a route to prioritize in future efforts [235]. Vitamin E and high doses of beta-carotene have been shown to increase prostate and lung cancer risk suggesting that caution is warranted with these powerful biological components [236], [237]. Recently it has become evident that the function of healthy tissues also relies on the activity of angiogenic factors. For example, currently used anti-VEGF drugs cause a marked reduction in vascular density, with a subsequent decline in the function of healthy organs such as the thymus and other endocrine glands, intestine and other parts of the digestive tract, gonads and the kidney [238]. As such, the effects that we are seeking from the treatment – potent and broad-acting anti-angiogenic activity – may end up leading to side-effects. Possible ways to overcome this issue is by targeted delivery such as via nanoparticle carriers as discussed above, or by titrating the amount of phytochemicals given such that it is effective in reducing/preventing pathological angiogenesis but not high enough to impair healthy vascular functions. Such an approach could logically be considered together with the combination of for example the 10 phytochemicals identified here in one cocktail where each compound is selected to complement the other hopefully leading to synergistic anti-angiogenic effects while maintaining negligible toxicity. This concept, as well as the identification of which tumor types are more susceptible to such treatment and identification of readouts for efficacy and toxicity, are important areas for future research.

There are, however, also potential drawbacks associated with the use of phytochemicals, which needs to be resolved before effective formulations of such compounds can be achieved. Due to poor absorption of many phytochemicals in their natural form, it may be necessary to chemically modify some of the compounds suggested here, or use them in semi-synthetic formulations such as nanoparticle encapsulations, which would lower their “natural” status but could promote their therapeutic and pharmacokinetic profiles. How best to take advantage of such derivations or modifications is still an open question.

## 8 Final conclusion

The complex and multi-factorial nature of tumor angiogenesis, especially in advanced tumors, necessitate the use of therapeutic compounds which act broadly in order to reduce problems with developing resistance. Cancer is a chronic disorder, which in cases where no curative treatment exists, has to be treated medically for a long period of time. Therefore treatment options with very low if any toxicity are preferred. Cancer is furthermore a growing medical issue also in developing countries and in societies in which the high costs associated with cutting edge treatment at modern medical institutions is prohibitive to the majority of the patients. Therefore low-cost treatment options have great potential. Phytochemicals constitute a class of broadly acting and very cheap natural drugs with very low toxicity especially if given at relatively low doses in combination. The list of 10 prototypical phytochemical approaches to inhibit 10 important pro-angiogenic targets in cancer presented here, constitute a framework for further studies on how to mix a potentially effective, non-toxic cocktail of natural compounds as a complement or alternative to other types of anti-cancer drugs available today. We hope that this review could serve as a primer for such investigations, which hopefully could lead to cheaper, safer and more effective anti-cancer treatment in the future.

## Conflict of interest statement

The authors declare no conflict of interest.

## Author contributions

Z.W., C.D., X.Y., M.F., A.A., W.K.R., D.G., G.R.N., B.E.R., D.R., Y.C.C. and L.D.J. wrote the manuscript, K.H., H.F., A.G.G., S.H., A.A., E.N., A.A., S.S.A., B.H., X.Y., G.G., D.B., M.R.C., K.A., S.C., D.H., S.I.M., A.A., A.B. and N.K. provided validation of the prioritized targets and approaches listed in Table 1, the result of which is shown in Table 1, Table 2.

## Figures and Tables

**Table 1:** Effects of the selected targets for anti-angiogenic cancer therapy on other cancer “hallmarks”.

Other cancer hallmarks	Angiogenesis targets									
	(Inhibit) endothelial cell migra-tion/tip cell formation	(Reduce) structural abnormalities of tumor vessels	(Reduce) hypoxia	(Inhibit) lymphangiogenesis	(Reduce) elevated interstitial fluid pressure	(Reverse) poor perfusion	(Norma-ize) disrupted circadian rhythms	(Suppress) tumor promoting inflammation	(De-activate) tumor promoting fibroblasts	(Normalize) tumor cell metabolism/acidosis
Genetic instability	0	0	+ [239]	0	0	0	+ [240]	0	0	+ [241]
Sustained proliferative signaling	0	0	+ [242]	+/− [243], [244]	+ [245], [246]	0	+ [247], [248]	0	+ [249], [250]	0
Tumor-promoting inflammation	+ [251]	+ [252]	+ [253], [254]	0	+ [255]	+ [252], [256]	+ [257]	NA	+ [258]	+ [259]
Evasion of anti-growth signaling	+ [260]	0	+/− [261], [262]	0	+ [246]	+/− [263], [264], [265]	+/− [266], [267]	+ [268]	+ [269], [270]	+ [271], [272]
Resistance to apoptosis	0	+ [273]	+ [274]	0	+ [275]	+ [276]	+ [277]	+ [278]	+ [279]	+ [280]
Replicative immortality	0	0	+ [281], [282], [283]	0	0	0	+/− [284], [285]	0	0	0
Dysregulated metabolism	+ [286]	+ [287], [288]	+ [289], [290], [291]	0	+ [292]	+ [293], [294]	+ [295]	0	+ [296]	+ [297]
Immune system evasion	+ [298]	+ [299], [300]	+ [301], [302]	+ [303]	+ [304], [305]	+ [306]	+ [307]	+ [308], [309]	+ [310], [311]	+ [312], [313]
Invasion and metastasis	+ [287]	+ [15], [314]	+ [315]	+ [316]	+ [317], [318], [319]	+ [320]	+ [321], [322], [323]	+ [324]	+ [323]	+ [325], [326]
Interactions in the tumor micro-environment	+ [260]	+ [327]	+ [328]	+ [329]	+ [330]	+ [327]	+ [331]	+ [332]	+ [333], [334]	+ [335]

Our 10 identified targets of anti-angiogenesis therapy are presented in the top row. 10 other cancer “hallmarks” are listed in the column to the left. Positive interactions (i.e. if the anti-angiogenesis target could also be a target for the indicated “hallmark”) are denoted “+”, controversial interactions (i.e. if the anti-angiogenesis target could both promote and inhibit the indicated “hallmark”) are denoted “+/−” and no interaction (i.e. if we have not been able to find any interaction between the anti-angiogenesis target and the indicated “hallmark”) is denoted “0”.

**Table 2:** Effects of phytochemical approaches in anti-angiogenic therapy as effective also against other cancer “hallmarks”.

Other cancer hallmarks	Phytochemical approach									
	Oleanolic acid	Tripterine	Silibinin	Curcumin	Epigallo-catechin-gallate (EGCG)	Kaempferol	Melatonin	Enterolactone	Withaferin A	Resveratrol
Genetic instability	+ [336]	0	+ [337]	+ [338]	+/− [339], [340]	0	+ [341]	0	0	+ [342]
Sustained proliferative signaling	0	0	+/− [343]	0	+ [245], [246]	0	+ [344], [345]	+ [346]	+ [249], [250]	+ [347], [348]
Tumor-promoting inflammation	+ [20]	0	+ [349]	+ [350], [351]	+ [352], [353]	+ [169]	0	+ [354]	0	+ [355], [356], [357]
Evasion of anti-growth signaling	+ [341], [358], [359]	+ [360], [361], [362]	+ [363], [364], [365], [366]	+ [367], [368], [369], [370]	+ [371], [372], [373], [374]	+ [375], [376], [377], [378]	+ [379]	+ [380]	+ [381], [382], [383]	+ [384], [385], [386], [387]
Resistance to apoptosis	+ [388], [389]	+	+ [390]	+ [391]	+ [374], [392], [393]	+ [394]	+ [395]	+ [396]	+ [397]	+ [398]
Replicative immortality	+ [399], [400]	0	+ [401], [402], [403]	+ [401], [404]	+ [405], [406], [407]	0	+ [408]	0	0	+ [409]
Dysregulated metabolism	+ [358], [410]	0	+ [411], [412]	+ [391], [392], [413]	+ [414], [415], [416]	+ [375], [417], [418]	+ [419], [420], [421]	0	+ [422], [423], [424]	+ [425], [426], [427]
Immune system evasion	+ [428], [429]	0	+ [430]	+ [431]	+ [432], [433]	0	+ [434]	0	+ [435]	+/− [436], [437], [438]
Invasion and metastasis	+ [439], [440]	+ [441]	+ [442]	+ [443]	+ [444], [445], [446]	+ [447], [448]	+ [449], [450]	+ [451]	+ [452]	+/− [453], [454], [455]
Interactions in the tumor micro-environment	+ [456]	+ [457]	+ [458]	+ [459]	+ [433], [460]	+ [461], [462]	+ [331], [463]	+ [199]	+ [464]	+ [465]

Our 20 identified therapeutic approaches for anti-angiogenesis therapy are presented in the top row. 10 other cancer “hallmarks” are listed in the column to the left. Positive interactions (i.e. if the compound could also exhibit therapeutic potential against the indicated “hallmark”) are denoted “+”, controversial interactions (i.e. if the compound could both promote and inhibit the indicated “hallmark”) are denoted “+/−” and no interaction (i.e. if we have not been able to find any therapeutic activity of the compound against the indicated “hallmark”) is denoted “0”.
